# A Genetic Basis of Susceptibility to Acute Pyelonephritis

**DOI:** 10.1371/journal.pone.0000825

**Published:** 2007-09-05

**Authors:** Ann-Charlotte Lundstedt, Shane McCarthy, Mattias C.U. Gustafsson, Gabriela Godaly, Ulf Jodal, Diana Karpman, Irene Leijonhufvud, Carin Lindén, Jeanette Martinell, Bryndis Ragnarsdottir, Martin Samuelsson, Lennart Truedsson, Björn Andersson, Catharina Svanborg

**Affiliations:** 1 Department of Microbiology, Immunology and Glycobiology, Institute of Laboratory Medicine, Lund University, Lund, Sweden; 2 Department of Cell and Molecular Biology, Karolinska Institutet, Stockholm, Sweden; 3 Department of Pediatrics, the Queen Silvia Children's Hospital, Gothenburg University, Gothenburg, Sweden; 4 Department of Pediatrics, Lund University, Lund, Sweden; 5 Department of Infectious Diseases, Gothenburg University, Gothenburg, Sweden; National Institute on Aging, United States of America

## Abstract

**Background:**

For unknown reasons, urinary tract infections (UTIs) are clustered in certain individuals. Here we propose a novel, genetically determined cause of susceptibility to acute pyelonephritis, which is the most severe form of UTI. The IL-8 receptor, *CXCR1*, was identified as a candidate gene when mIL-8Rh mutant mice developed acute pyelonephritis (APN) with severe tissue damage.

**Methods and Findings:**

We have obtained *CXCR1* sequences from two, highly selected APN prone patient groups, and detected three unique mutations and two known polymorphisms with a genotype frequency of 23% and 25% compared to 7% in controls (p<0.001 and p<0.0001, respectively). When reflux was excluded, 54% of the patients had *CXCR1* sequence variants. The UTI prone children expressed less CXCR1 protein than the pediatric controls (p<0.0001) and two sequence variants were shown to impair transcription.

**Conclusions:**

The results identify a genetic innate immune deficiency, with a strong link to APN and renal scarring.

## Introduction

The genetic basis of susceptibility to common infectious diseases is largely not determined, except for one or two classical examples like malaria and hemoglobin A/E polymorphisms [1], [2]. Our laboratory has for many years been involved in an attempt to characterize the determinants of susceptibility to acute and chronic form of urinary tract infection (UTI) but so far, we and others have failed to identify genetic factors determining disease susceptibility in man. In an effort to characterize susceptibility mechanisms and gene(s) associated with particular infections, we have studied UTI susceptibility in “knock-out” mice with defects in specific loci [3], [5], . We have obtained evidence that the deletion of a single gene encoding the murine IL-8 chemokine receptor homologue (mIL-8Rh) precipitates the entire syndrome of acute pyelonephritis (APN) and renal scarring. In view of these findings, we have investigated whether genetic variability in the human chemokine receptor gene (*CXCR1*) might contribute to the disease incidence in APN-prone individuals.

UTIs are among the most prevalent bacterial infections in man and remain a significant concern due to their frequency and associated morbidity and mortality [6], [7]. Acute pyelonephritis (APN) is the most severe and rare form of UTI, and recurrent APN is clustered in a small group of highly susceptible individuals, some of whom develop progressive renal scarring and may need dialysis and transplantation [7], [8], [9]. There have been many attempts to identify the host factors, which predispose to UTI, and especially to APN. Mechanical dysfunctions like vesico-ureteric reflux increase the access of bacterial to the kidneys, but uro-dynamic abnormalities alone do not render patients prone to APN [8], [9], [10]. The P blood group and secretor state determine the mucosal repertoire of receptors for P fimbriae and help select the infecting *Escherichia coli* strain, but variant receptor expression does not influence the efficiency of the antibacterial defense [10]. Furthermore, Mendelian primary immuno-deficiencies do not predispose to UTI in man [11], [12] and attempts to relate the HLA antigen type to UTI have failed [13].

The urinary tract relies on innate immunity to eliminate clearance and maintain tissue integrity, and single gene defects have been shown to confer susceptibility in the murine model [14], [15]. The mIL-8Rh chemokine receptor mutant mice develop acute, septic pyelonephritis with about 50% mortality [4], [12], [16]. They lack the single chemokine receptor for neutrophil chemo-attractants and develop an exaggerated acute inflammatory response, which leads to renal scarring [4], [12], [16]. Based on the susceptibility of the mIL-8Rh mutant mice, we performed a preliminary clinical study of IL-8 receptor expression in APN prone children [5]. Two human receptors interact with IL-8 and related chemokines [17], [18]. CXCR1 is specific for IL-8 and GCP-2 while CXCR2 is more promiscuous [19]. We found that the expression of CXCR1 but not CXCR2 was reduced, suggesting that variant CXCR1 receptor expression might influence human APN susceptibility [5]. A recent family study showed a strong accumulation of APN, further suggesting that APN susceptibility might be inherited [20].

Here we have identified disease-associated polymorphisms and mutations in the *CXCR1* gene among APN prone patients, suggesting a novel, genetically determined cause of APN susceptibility.

## Materials and Methods

### Patients

Sixty patients with APN and recurrent UTI were studied. 24 infants and children were followed from their first episode of APN, with regular controls at the Department of Pediatrics, Lund University Hospital for a median of 4.5 years. They were <1 to 9 years old (median 1.5 years) at the first infection and 1 to 12 years old (median 6 years) at the time of testing (for clinical data, see Supporting Information Table S1). Seven patients had recurrent pyelonephritis, 23 had an initial episode of pyelonephritis followed by episodes of acute cystitis (n = 7) or asymptomatic bacteriuria (ABU, n = 4). Seven children had a single episode of APN. On 99mTc-DMSA scintigraphy, 19 of the children had renal polar uptake defects typical of pyelonephritis, nine children showed renal scars. All children underwent ultrasound investigation and voiding cystourethography (VCUG) and vesico-ureteric reflux (VUR) was detected in 11/24, of which one had structural abnormalities (double ureters), and one had ureterocele. Hydronephrosis was found in 2/24. The remaining 11 patients had no structural abnormalities (Table S1).

All but one pediatric patient were Caucasians, and 21 were born to Swedish parents. The father of P8 was from Slovakia, P13 was of Polish origin and P14 was adopted from China. Variants 1 and 2 were detected in patients P8, P13 and P14, but the remaining patients with variants 1 and 2 and those with variants 3, 4 and 5 were born to Swedish parents.

Thirty-six patients were adults with a history of childhood APN, who participated in a study of febrile UTI in the 1970ies (median age 4 years) and were followed regularly since then. Between 2002 and 2005, a median of 30 years after the initial UTI episode the patients were reinvestigated [21]. Samples for *CXCR1* analysis in this study were obtained, the UTI history was recorded and the kidney status was defined by DMSA scans and Cr51 EDTA clearance. All adult patients were Caucasians.

Significant bacteriuria was defined by growth of a single strain (>10^5^ cfu/ml) in a mid-stream urine sample, or by any growth in a supra-pubic bladder aspirate. Pyelonephritis was defined as a febrile infection (≥38.5°C) with significant bacteriuria, C-reactive protein >20 mg/l and lack of symptoms of other infections. ABU was defined as >10^5^ cfu/ml in three consecutive urine samples in an asymptomatic individual.

The studies were approved by the Medical Ethics Committees (IRBs) of the Lund University and the Gothenburg University (LU 236-99, LU 106-02). Informed consent was obtained from all subjects and/or the parents. Patient information was handled according to the HIPPA.

### Controls

Pediatric controls (n = 26) were enrolled when they attended the Pediatric outpatient clinic or were admitted for elective surgery and were interviewed to ensure that they had no history of UTI or other severe infections. There were 26 children (15 boys and 11 girls) aged 1 to 13 years at the time of sampling (median 6 years). CXCR1 expression was examined in 16 and *CXCR1* variants in 26 controls. Adult healthy blood donors (n = 200) from the same geographic area were included as controls to assess the frequency of *CXCR1* sequence variants in the background population. Their UTI history had not been penetrated. The pediatric controls were born to Swedish parents.

### Genomic DNA Analysis

Genomic DNA was extracted from peripheral blood neutrophils using proteinase K and phenol-chloroform. Specific primer pairs were designed according to the published genomic DNA sequence for *CXCR1* (GenBank accession number: L19592). Primers were chosen based on 3′ specificity for the *CXCR1* gene (Table S2), in order to avoid mis-amplification of the *CXCR1* pseudo-gene (gi 186372). Patient forward and reverse sequences were base called and multi-aligned along with control sequences using PolyPhred [22] and Phrap (http://www.phrap.org) respectively.

Sequencing of the amplicons was in both directions, using specific nested primers covering 800 bp upstream of the transcription start to 117 bp downstream of the poly-adenylation (PolyA) signal of the larger of the two mRNA transcripts [23]. In total 5119 bps were amplified from each individual DNA (not including overlaps), resulting in 255.45 kbp for sequence analysis, comparison and single nucleotide polymorphism (SNP) mining. In addition, 1261 bp encompassing a 500 bp fragment upstream of the gene was sequenced to examine possible additional regulatory elements. The sequences were visualized and manually compared using Consed [24]. Known SNPs in dbSNP [25] were tagged to identify novel variants. The *CXCR1* promoter (nt −800 to +98) was sequenced using the BigDye™ Terminator Cycle Sequencing v2.0 Ready Reaction Kit and a fluorescence based automated cycle sequencer, ABI PRISM™ 377 (Perkin-Elmer Applied Biosystems) Data were analyzed using BioEdit (T. Hall, http://www.mbio.ncsu.edu/BioEdit/bioedit.html). The *CXCR1* gene region (nt −3342 to −2071 and nt −580 to +4318) was sequenced on a MegaBACE 1000 using the DYEnamic™ ET dye Terminator Kit (Megabace™) (Amersham Pharmacia Biotech). Data were analyzed using Polyphred–Phrap and Consed.

### Pyrosequencing

Variants in *CXCR1* were identified in a Pyrosequencer PSQ 96 using the PSQ 96 SNP Reagent Kit (Pyrosequencing AB, Uppsala, Sweden). The PCR amplification primers for variants 1–5, the nested PCR and pyrosequencing sequencing primers (Table S3) were designed according to the manufacturer's instructions (Pyrosequencing AB; http://www.pyrosequencing.com).

### CXCR1 receptor expression

Neutrophils were purified from heparinized whole blood on a Polymorphprep™ density gradient (AXIS-SHIELD, PoC AS) and surface expression of CXCR1 was detected by confocal microscopy (Bio-Rad Laboratories) and quantified by flow cytometry (Coulter, 3000 cells/sample) as previously described [5]. Receptor expression in patient and control samples was related to an adult standard, run at the same time [5].

### Protein extracts and Electrophoretic Mobility Shift Assay (EMSA)

Nuclear extracts were prepared from the myeloid cell-line HL60, clone 15, (ATCC No. CRL-1964) [26] with 0.6% NP-40 in the lysis buffer and protease inhibitors (Complete, Roche) in all buffers [27], and stored as aliquots at −80°C. Protein concentrations were measured using the DC Protein Assay kit (Bio-Rad) with bovine serum albumin as standard. EMSA was performed using the Gel Shift Assay System (Promega). Double stranded oligonucleotides encompassing the putative RUNX1 binding site in the *CXCR1* intron (common allele 5′-CTCTTGTGACCACCACTCAT-3′; SNP1 5′-CTCTTGTGACCAGCACTCAT-3′) were end-labeled with [γ-^32^P]ATP (Amersham Biotech) to similar specific activities. DNA-protein complexes were separated on 6% polyacrylamide TBE gels (Invitrogen) and visualized by autoradiography in a PhosphorImager, STORM 840 (Amersham Pharmacia Biotech). Unlabeled ds-oligonucleotides at 10- to 100-fold molar excess were used in a competition assay with oligonucleotide 5′-TTGAACGTCACATCTTTAAC-3′ as an unspecific competitor, and quantified in a PhosphorImager, STORM 840 (Amersham Pharmacia Biotech). The DNA-binding protein was identified using a RUNX1-specific antibody (AML-1, sc-8563 X) or an irrelevant antibody control (ATF-2, sc-6233 X) (Santa Cruz Biotech). The TFSEARCH database was used to predict the transcription factor binding sites. (http://molsun1.cbrc.aist.go.jp/research/db/TFSEARCH.html) [28].

### Real-time PCR

Total RNA was reverse transcribed using the TaqMan Reverse Transcription Reagents kit and random hexamers or oligo dT primers according to the manufacturer's instructions (Applied Biosystems). Residual genomic DNA was removed using RQ1 RNase-free DNase (Promega). GAPDH (Assay ID Hs99999905_ml, Applied Biosystems), CXCR1 total (assay ID Hs00174146_ml, Applied Biosystems) and CXCR1 large (specifics, see below) transcripts were quantified by real-time PCR using a Corbett Research Rotor-Gene instrument. The assay was designed using Vector NTI (Informax), CXCR1 forward primer 5′-GGTTGTGACAGAGTCAAGGGTGTGT-3′ and reverse primer 5′-TGTGCCTCAAGAGACTGTTCTAGCA-3′. The probe 5′-GGCAGCACCTCC TAAGAAGGCA CCT-3′ was 5′-end labeled with FAM and 3′-end labeled with Black Hole Quencher 1 (MWG Biotech).

### Luciferase reporter assay

We constructed luciferase reporter plasmids by cloning a single copy of the RUNX1 binding motif containing the wild-type (pAML1wt-TK-luc) or the SNP1 (pAML1SNP1-TK-luc) allele upstream of the TK promoter. The plasmids were constructed by cloning the annealed 5′-phosphorylated primer pairs in pGL3-TK-luc vector cleaved with *Xho*I and *Bam*HI. For primers see Table S3.

For the luciferase assay, we cultured A498 cells in RPMI 1640 medium supplemented with 10% fetal bovine serum in 6-well culture plates at a density of 5×10^5^ cells per well. The cells were transiently transfected with 3.5 µg of either constructs and co-transfected with 0.10 µg of a plasmid encoding AML-1b [27], (kindly provided by Dr. U. Gullberg, Lund University, Lund, Sweden) by using Lipofectamine 2000 (Invitrogene). A reporter construct (3,5 µg) containing the *CXCR1* promoter, exon 1, the intron with a RUNX1 binding motif and exon 2 up to the coding sequence were transiently transfected into the A498 cells. The cells were also co-transfected with 0.10 µg of the plasmid encoding AML-1b and/or with 0.10 µg of a plasmid encoding PU.1 [27], (kindly provided by Dr. U. Gullberg, Lund University, Lund, Sweden). The cells were collected 24 h post-transfection and luciferase activity was measured with the Dual-Luciferase Reporter Assay System (Promega).

### Statistical Analysis

CXCR1 expression and mRNA levels in pediatric patients and controls and the luciferase assay data was compared by the Mann-Whitney *U* test, two-sided. The *CXCR1* genotype and allele frequency was examined using chi-square Test. The Fishers' exact test was used to calculate the total *CXCR1* frequency of in patients with and without VUR compared to pediatric controls. (GraphPad Instat 3 for Macintosh (GraphPad Softwear, Inc.).

## Results

The different forms of UTI must be distinguished, to appreciate differences in disease susceptibility and to enrich for low frequency genetic factors. The patients in the present study were a subset of children with APN, which is the most severe but least frequent form of UTI. The cumulative APN frequency is about four per cent up to 7 years of age, and only about 1/100 to 1/200 of patients experience recurrent APN after a first APN episode [29], [30]. As a consequence, children with APN and recurrent UTI represent a highly selected subset of all patients with childhood UTI.

Here, we have examined *CXCR1* DNA sequences in two, independent groups of APN prone individuals. The first group consisted of prospectively enrolled children (n = 24) who were followed from their first episode of APN, with regular controls at the Department of Pediatrics, Lund University Hospital. They were <1 to 9 years old (median 1.5 years) at the first infection and 1 to 12 years old (median 6 years) at the time of testing (for clinical data, see Table S1). Pediatric controls of the same age (n = 26) were enrolled when they attended the Lund university hospital for diagnoses unrelated to infection.

The second patient group was enrolled in a prospective study of febrile UTI in the 1970ies at a median age of 4 years and was prospectively followed [21]. The patients were reinvestigated between 2002 and 2005, a median of 30 years after the initial APN episode, and samples for *CXCR1* sequencing were obtained from 36 patients, who had a history of APN and recurrent UTI. Adult healthy blood donors (n = 200) were included, to assess the frequency of *CXCR1* sequence variants in the background population.

### *CXCR1* sequence variants

**Figure 1:**
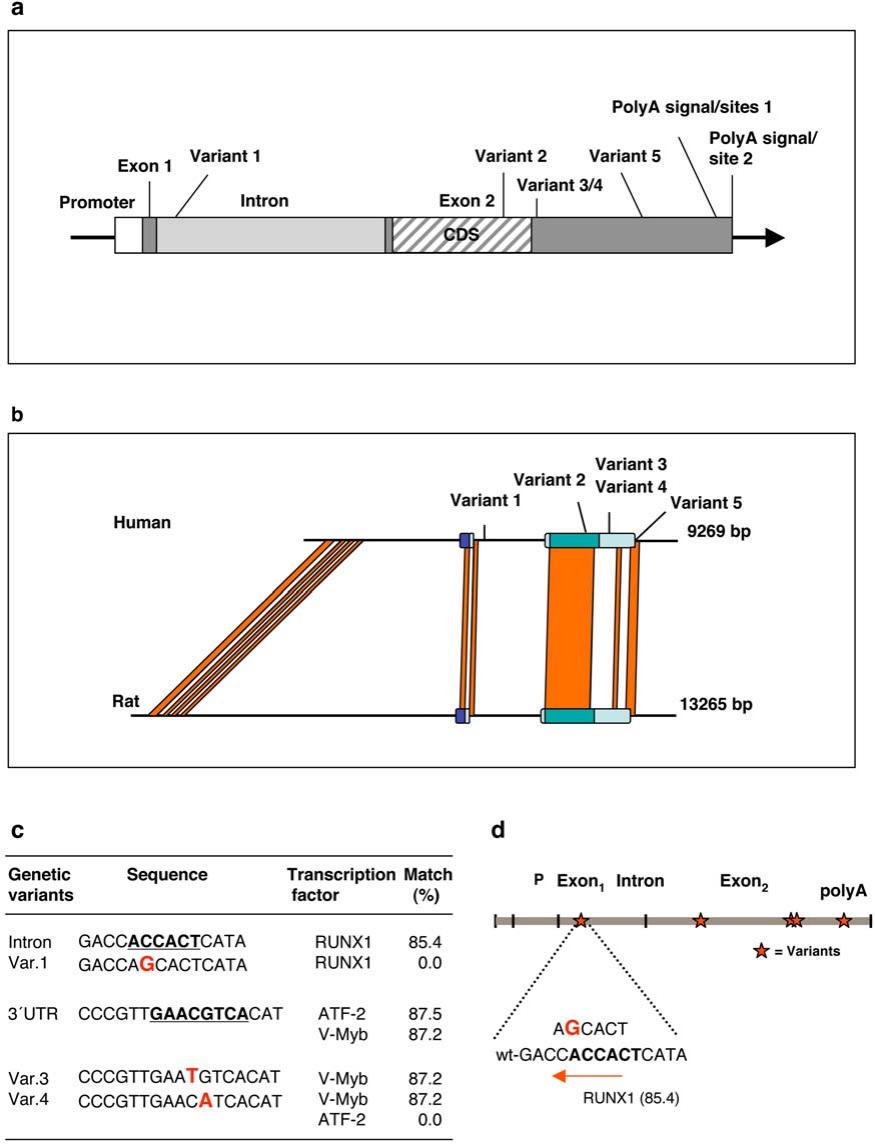
Sequence variation in the human CXCR1 locus. a, Genomic organization of *CXCR1* and positions of genetic variants (Variants 1–5) in the intron, the coding sequence (CDS) and in the 3′untranslation region (3′UTR). b, Comparison of the human *CXCR1* gene with its rat ortholog (*Il8ra*). BLAST homologies indicating strongly conserved regions are shown by the lines connecting the genes. The five *CXCR1* variants identified in the patients are all located in or near strongly conserved regions as indicated. The rat was selected, as the human *CXCR1* gene does not have an ortholog in the mouse where the equivalent of the human CXCR2 (mIL-8Rh or *Il8rb*) gene carries out the same function. c, Predicted effects of *CXCR1* variants on putative transcription factor binding motifs based on a TRANSFAC search. The transcription factors and % score match of the mutant alleles are shown. d, Variant 1 (enlarged, bold) potentially disrupts the RUNX1 binding motif.

The human IL-8 receptor genes *CXCR1* and *CXCR2* and a homologous pseudo-gene have been mapped to position 2q35 [31], [32]. *CXCR1* comprises two exons interrupted by an intron of 1.7 kb and the entire coding sequence is in exon 2 (Fig. 1a). The *CXCR1* promoter (−841 to +21) contains a TATA box equivalent, GC-rich motifs that may serve as SP-1 and AP-2 sites, and a GGAA motif serving as a binding site for PU.1, which is a member of the Ets family of transcription factors. Most of the promoter activity is determined by sequences from −56 bp to +50 bp, relative to the transcription start site, with positive regulatory elements located at −126 to +50 bp, and negative regulatory elements located upstream from −126 to about −640 bp. The GGAA binding site for PU.1 is adjacent to the transcription start site at −7 to −4 [23], [33], [34].

Genomic DNA was sequenced using overlapping primers, covering the entire *CXCR1* gene (Fig. 1a, Table S2). The nucleotides were numbered relative to their distance from the transcription start site. Five sequence variants were detected in the intron, the coding region of exon 2 and in the 3′ untranslated region (3′UTR) of *CXCR1* (Fig. 1a). Variant 1 in the intron was a C to G nucleotide substitution at position +217 (3943:L19592), 217 bp from the transcription start site and 317 bases upstream of an ALU element. Variant 2 in exon 2 was a G to C substitution at position +2608 (6334:L19592), resulting in a non-synonymous amino acid change from Serine (Ser) to Threonine (Thr). In the 3′UTR, a C to T transition (variant 3) was detected at position +3081 (6807:L19592) and a G to A transition (variant 4) at position +3082 (6808:L19592). Variant 5 was a G to A transition at position +3665 (7391:L19592) between the Poly(A) signal and the Poly(A) sites of the short mRNA (Fig. 1a).

### Difference in SNP frequency between patients and controls

Full-length CXCR1 DNA sequences were obtained from 12 pediatric patients, 35 adult patients with childhood APN and 12 pediatric controls without a history of UTI. In addition, 12 pediatric patients, 1 adult patient, 14 pediatric controls and DNA from 200 adult healthy blood donors was screened for the identified *CXCR1* variants, using pyrosequencing with primers specific for each variant.

Single base changes in *CXCR1* were associated with APN susceptibility in the pediatric population. Sequence variants were detected in 9/24 (37.5%) of the APN prone children, but only in 1/26 (4%) of the pediatric controls (Table 1). Six patients were heterozygous for variants 1 and 2, which were present on 6/48 chromosomes, resulting in minor allele frequencies of 13%. One control child was homozygous for variant 1, resulting in a minor allele frequency of 4%, (2/52 chromosomes). Sequencing of parent DNA showed that variants 1 and 2 were linked. Variants 3, 4 and 5 occurred as heterozygous mutations in one patient each and were unique for the pediatric patients.

**Table 1:** Number of individuals with CXCR1 sequence variants

	Variant sequences	Consensus sequence	Total
	n (%)	n (%)	n
**APN in childhood**	**19 (32)**	**41 (68)**	**60**
-Lund population	9 (37.5)	15 (62.5)	24
-Gothenburg population	10 (28)	26 (72)	36
**Controls**	**17 (8)**	**209 (92)**	**226**
-Pediatric	1 (4)	25 (96)	26
-Adult	16 (8)	184 (92)	200

APN = Acute pyelonephritis. Lund population = Paediatric patients followed from their first infection. Gothenburg = patients with APN in childhood during the 1970ies who were re-examined for this study.

In the adults, *CXCR1* variants 1 and 2 were detected in 9/36 (25%) and 10/36 (28%), respectively. One patient was homozygous for variant 2, resulting in a minor allele frequency of 15% for variant 2 and 13% for variant 1 in this group. Variants 1 and 2 were detected by pyrosequencing in 16/200 (8%) of controls on 16/400 chromosomes, resulting in a minor allele frequency of 4% (Table 1, p = 0.0056 for variant 1 and p = 0.0018 for variant 2 compared to the adult patients and p = 0.0185 compared to the children with APN, Fischer's exact test, two-sided). The results showed that single base changes in *CXCR1* are associated with susceptibility to APN also in adults, thus confirming the disease association of these variants (Table 2, Table 3).

**Table 2:** Frequency of CXCR1 sequence variants 1 and 2

	Genotype frequency, variant 1 (+217)^#^				Genotype frequency, variant 2 (+2608)^##^			
	CC (%)	Cg (%)	gg (%)	p^a)^	GG (%)	Gc (%)	cc (%)	p^b)^
**APN total n = 60**	45 (75)	14 (23)	1 (2)	0.0007	44 (73)	15 (25)	1 (2)	<0.0001
**Control total n = 226**	209 (92.5)	16 (7)	1 (0.5)		210 (93)	16 (7)	0	

APN = Acute pyelonephritis; n = number of individuals; ^#^ nucleotide at position +217 in the intron. ^##^ nucleotide at position +2608 in the coding sequence. ^a), b)^ = Chi-square Test, total number of APN prone patients versus total number of controls.

**Table 3:** Allele frequency of CXCR1 sequence variants 1 and 2

	Allele frequency (+217)^#^			Allele frequency (+2608) ^##^		
	C (%)	g (%)	p^a)^	G (%)	c (%)	p^b)^
**APN prone, total n = 60** N = 120	104 (87)	16 (13)	0.0007	103 (86)	17 (14)	<0.0001
**Control total n = 226 N = 452**	434 (96)	18 (4)		436 (96)	16 (4)	

APN = Acute pyelonephritis; n = number of individuals; N = number of alleles; ^#^ nucleotide at position, +217 in the intron. ^##^ nucleotide at position +2608 in the coding sequence. ^a), b)^ Fischer's Exact Test, total number of APN prone patients versus total number of controls.

### Putative effects on CXCR1 expression

Variants 1, 3 and 4 were located to sequences with high homology to transcription factor binding motifs, identified by TRANSFAC (Fig. 1c). Variant 1 (217C/G) was in a putative binding site for the runt-related transcription factor 1 (RUNX1, also called AML1) (Fig. 1d), which is required for expression from a number of cell specific enhancers and promoters [35], [36]. The cyclic-AMP-dependent transcription factor ATF-2 (CRE-BP1, compatible transcription factor motif of the common allele) was lost in both the variant 3 (+3081T) and variant 4 (+3082A) -bearing alleles but they retained the potential v-Myb DNA-binding sites from the common allele (Fig. 1c).

**Figure 2:**
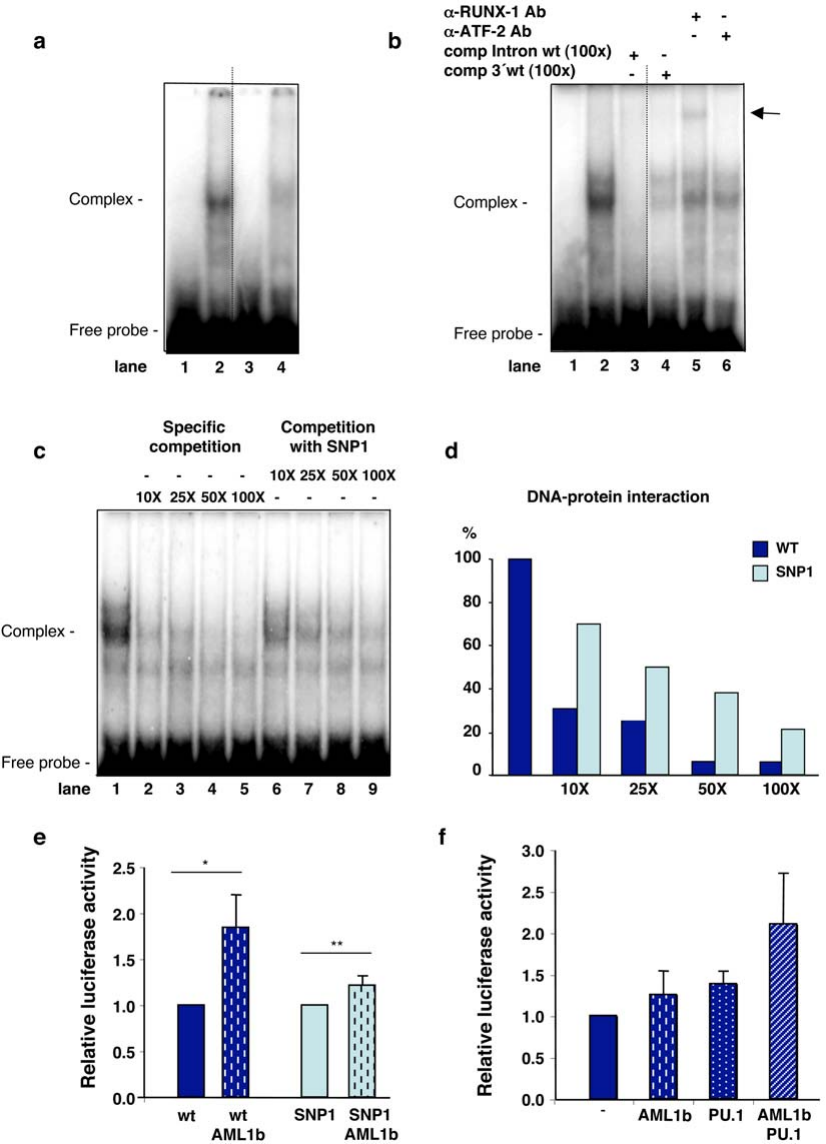
Effects of CXCR1 sequence variants on transcription. a, EMSA, showing the binding to the putative RUNX1 oligonucleotides of proteins in a HL60 cell nuclear extract. Binding (arrow) was stronger to the wild type (lane 2) than to the variant 1 oligonucleotide (lane 4). Lanes 1 and 3 are probes without proteins. b, Competitive inhibition of binding by cold intact probe (100-fold excess, lane 3), but not by unspecific probe (100× excess, lane 4). A super-shifted band, indicated by the arrow, was obtained with anti-RUNX1 (lane 5) but not with control antibody (anti ATF-2) (lane 6). Hatched lines indicate removed excess lanes. c, Inhibition of specific wild-type binding by unlabeled wt probe (10×–100×, lanes 2–5) and reduced efficiency of competition with unlabelled SNP1 probe (10×–100×, lane 6–9). d, The inhibition of the DNA-protein interaction in Panel *c* was quantified in a Phosphor Imager. e, Effect of SNP1 on RUNX1-dependent transcriptional transactivation. Allelic differences in relative luciferase activity in pAML1 (wt/SNP1)-TK-luc transfected A498 cells with or without co-transfection with an AML-1b expression vector. Data show the mean ± SEMs of three separate experiments done in duplicate. *P = 0.0104 and **P = 0.1199 by the Mann-Whitney *U* test, two-tailed. f, RUNX1 and PU.1 interacts with the *CXCR1* promoter in transfected A498 cells. The *CXCR1* promoter activity was quantified using luciferase. The signal was enhanced by co-transfection with the AML1b (RUNX1) and PU.1 expression plasmids.

The reduction in RUNX1 binding to the variant 1 sequence was confirmed by electrophoretic mobility shift assay (EMSA) (Fig. 2). Nuclear extracts from the HL60 cell-line were used. HL60 cells are promyelocytic leukemic cells and a significant percentage of the cultured cells (10–12%) differentiate spontaneously into mature neutrophils [37]. The HL60 cells are thus neutrophil like and are an accepted model to study neutrophil cells. In addition, they express high amounts of the transcription factor AML1. Mature neutrophils, in contrast, are end stage cells, which are difficult to maintain *in vitro* for more than a few hours and which are difficult to transfect. Furthermore, the concentration of proteolytic enzymes is very high and it is difficult to isolate intact nuclear proteins.

HL60 cell nuclear extracts were incubated with oligonucleotide probes encompassing the putative RUNX1 binding site, and complex formation was detected by gel electrophoresis. There was a significant decrease in RUNX1 binding to the mutated sequence compared to the common allele (wt-probe) (Fig. 2a, lanes 2 and 4). The specificity was confirmed by competition with cold common allele probe at 100 fold molar excess, but an unrelated probe had no effect (Fig. 2b, lane 3 respective 4). The bound protein was identified as RUNX1 by specific antibody (Fig. 2b, lane 5) in a super-shift assay and the specificity was confirmed by competitive inhibition with unlabelled wt-probe (Fig. 2c, lane 2–5) or SNP1 (variant 1) probe (Fig. 2c, lane 6–9). Variant 1 was a less efficient inhibitor than the common allele (Fig. 2d).

To examine the effect of variant 1 on RUNX1 transcriptional transactivation, we constructed a luciferase reporter carrying the putative RUNX1 binding motif with the variant 1 (pAML1SNP1-TK-luc) or the wild type allele (pAML1wt-TK-luc) and the luciferase activity was used as a read out of RUNX1-dependent transcription. To control transcription, we selected human kidney epithelial cells (A498), which lack the RUNX1 transcription factor. RUNX1 dependent transcription was artificially induced, by co-transfecting the cells with an AML-1b expression vector and as a result, RUNX1 dependent transcription was enhanced (Fig. 2e). In this RUNX1 dependent assay, the luciferase activity from the mutant allele was reduced compared to the wild type allele, thus supporting the hypothesis that variant 1 reduces *CXCR1* transcription. Further evidence of RUNX1 involvement in *CXCR1* transcription was obtained by co-transfection of A498 cells with a *CXCR1* promoter reporter plasmid and vectors encoding RUNX1 and PU.1. The promoter was fully functional, and both RUNX1 and PU.1 were required to enhance luciferase activity (Fig. 2f).

### Aberrant CXCR1 mRNA processing associated with variant 5

*CXCR1* has two alternative poly(A) sites and a long and a short transcript are formed [23]. Variant 5 caused a 3′ G to A transition at position +3665, between the first poly(A) signal and the poly(A) sites (Fig. 1a, b). A similar polymorphism in the prothrombin gene has been shown to increase the efficiency of mRNA 3′-processing [38], [39]. If variant 5 had a similar effect, the levels of the long CXCR1 transcripts would be reduced. The long and total CXCR1 transcripts were quantified by RT-PCR using cDNA reverse transcribed with random hexamers or oligo dT primers. The mRNAs from the patient carrying variant 5 and the mother with the same mutation contained reduced levels of the CXCR1 large transcript compared to the control, thus confirming the predicted effect of variant 5, suggesting that this mutation might create a more efficient cleavage site and thus reduce the amount of large CXCR1 mRNA (data not shown).

### Vesico-ureteric reflux

Reflux is known to predispose to acute pyelonephritis and renal scarring. The relative contribution to APN susceptibility of vesico-ureteric reflux (VUR) and *CXCR1* sequence variation was therefore examined in the APN prone children, where 11/24 had VUR and two had structural abnormalities (ureterocele and double ureters) (Table 4). The patients without VUR had a higher frequency of *CXCR1* sequence variants (7/13, 54%) than the children with VUR (2/11, 18%, Table 4). The group without VUR was significantly different from the controls (p = 0.0007) but the patients with VUR were not (n.s., Table 4). The results suggest that *CXCR1* sequence variation and VUR are independent risk factors in APN-prone patients.

**Table 4:** CXCR1 sequence variants related to reflux

	Number (%) of individuals with variants					
	Var. 1	Var. 2	Var.3	Var.4	Var.5	Total+
APN prone children without VUR	4 (31)*	4 (31)**	1 (8)	1 (8)	1 (8)	7 (54)≠
APN prone children with VUR	2 (18)†	2 (18)‡	0	0	0	2 (18)
Pediatric controls	1 (4)	0	0	0	0	1 (4)

The frequency of variants was significantly higher in APN prone patients without VUR than in paediatric controls.

* p = 0.0345 for variant 1 in patients without VUR compared to pediatric controls

** p = 0.0087 for variant 2 in patients without VUR compared to pediatric controls

† p = 0.2053 for variant 1 in patients with VUR compared to pediatric controls

‡ p = 0.0826 for variant 2 in patients with VUR compared to pediatric controls

≠ p = 0.0007 for total variants in patients without VUR compared to pediatric controls

Fischeŕs exact test, two sided;

+ = number of individuals with variant *CXCR1* sequences

### Low CXCR1 surface expression in APN-prone children

**Figure 3:**
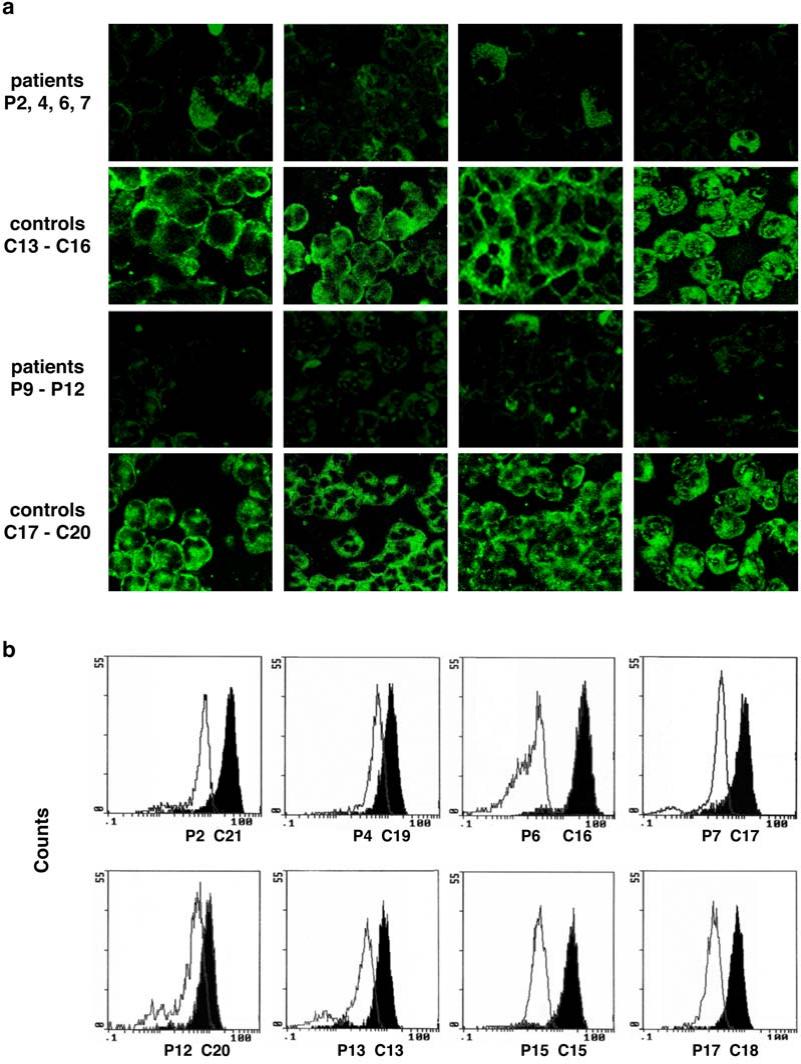
Low CXCR1 expression on neutrophils from pyelonephritis prone children compared to pediatric controls. a, Confocal microscopy images of individual samples from eight patient and controls, using a monoclonal anti-human CXCR1 primary and a FITC conjugated, anti-mouse secondary antibody. b, Quantification by flow cytometry of CXCR1 expression in patients (white peaks) and controls (black peaks).

We obtained peripheral blood neutrophils during an infection free interval or while the patient received antibiotic prophylaxis. By confocal microscopy (Fig. 3a) we observed that the surface staining for CXCR1 was markedly reduced in the patients compared to the controls. The difference in CXCR1 expression was quantified by flow cytometry analysis of 23 patient and 16 control samples (Fig. 3b). The patient CXCR1 expression showed a mean of −1,44, range −6.52–(1.04) compared to the standard while the controls showed a mean of 0.28, range −0.73–(2.15), (p<0.0001 for the group-wise analysis, Mann-Whitney *U* test, two-tailed). The results confirmed and extended data on CXCR1 expression from the preliminary study by Frendeus *et al*. [5]. Patients 1–12 in that study had CXCR1 levels of −1.33, range −6.52–(1.04). The newly recruited patients 13–21 and 23–24 in this study expressed a mean of −1.57, range −2.76–(0.63) compared to the standard.

## Discussion

A subset of all children with UTI are APN prone, and risk to develop recurrent infections and kidney damage [29]. There have been many attempts to identify the host factors, which predispose them to infection. Reflux, blood groups, social and environmental variables have been discussed but molecular markers have not been identified until mIL-8Rh mutant mice were shown to develop APN with bacteremia and renal scarring [6], [8], [9], [10]. Here we show that variation in the human IL-8 receptor gene may influence APN susceptibility and propose a mechanism how the variant alleles may suppress innate immunity in the urinary tract. Five *CXCR1* sequence variants were detected in carefully selected patients with APN. Three new 3′ mutations were unique to the patients and two known variants were more common in patients than in controls, supporting a disease association. Variant 1 reduced the efficiency of RUNX1 dependent transcription and another three variants were located to transcription factor binding sites. As expected, CXCR1 expression was markedly reduced in the APN prone children compared to controls. The results suggest that *CXCR1* variants may render individuals UTI-prone by lowering CXCR1 expression and by incapacitating the neutrophil-dependent host defense against UTI.

Innate immunity controls the resistance to UTI and neutrophils are needed for bacterial clearance from infected tissues. The chemokine receptor deficiency in mIL-8Rh knock out mice incapacitates neutrophils by delaying their migration into the kidneys and their exit from the tissues across the mucosal barrier. In addition, the receptor is needed for neutrophil activation and phagocytosis and killing of bacteria is impaired in knock out mice. As a result, bacteria persist in the kidneys, and the trapped neutrophils destroy the tissues [4], [12], [16]. Similar conclusions were drawn in a recent study, where polymorphisms in the *ICAM-1* gene were shown to protect against renal scarring following UTI by decreasing the number of neutrophils and thereby the inflammatory host response [40]. ICAM-1 is expressed on endothelial cells as well as on kidney and bladder epithelial cells and acts as a counter-receptor for Mac-1 [41]. ICAM-1 expression is increased on the cytokine-activated endothelium [42] and on infected epithelium [43] and is involved in the endothelial and epithelial transmigration of neutrophils [43], [44]. These observations illustrate the delicate balance between the protective and destructive aspects of the neutrophil-dependent defense against UTI.

The UTI associated *CXCR1* variant 1 was shown to reduce RUNX1 binding to the putative intronic binding site. Furthermore, transfection experiments showed that transcription of the mutant allele is reduced, suggesting that variant 1 reduces *CXCR1* transcription. RUNX1 activates transcription through protein-protein interactions with the Ets family of transcription factors, including PU.1, which is a regulator of CXCR1 expression [34], [36]. Neutrophils from PU.1-null mice fail to terminally differentiate and their neutrophils fail to respond to CXCL8, indicating that functional receptors are not expressed when this transcription factor is absent [45]. The importance of PU.1 for *CXCR1* transcription was supported by our *in vitro* transfection studies showing an interaction between transcription factors AML1b (RUNX1) and PU.1 promoted transcription in a luciferase reporter assay. Reduced RUNX1 binding caused by intronic SNPs has been proposed to cause aberrant regulation of *PDCD1* (the programmed cell death 1 gene) in patients with systemic lupus erythematosus [46], and of *SLC22A4* in patients with rheumatoid arthritis [47], [48]. In addition, increased susceptibility to psoriasis was associated with a loss of inter-genic RUNX1 binding [49].

The two additional 3′variants (3 and 4) were also proposed to influence transcription, based on TRANSFAC searches, which identified putative transcription factor binding sites. Variant 5 was associated with reduced levels of the large CXCR1 transcript, suggesting that this mutation might create a more efficient cleavage site and thus reduce the amount of large CXCR1 mRNA. A similar mutation was shown to create a more efficient mRNA cleavage site in the pro-thrombin gene where more efficient processing of the transcript leads to higher pro-thrombin levels and a higher risk of thrombosis [50]. In addition, there were several patients with low CXCR1 expression but without variation in the *CXCR1* gene. Thus, even if the frequency of patients with single base changes was high, there must be additional mechanisms, which control CXCR1 expression, and which may be polymorphic in this patient group.

Longitudinal clinical studies must be performed, to reliably identify those patients, who are susceptible to recurrent APN. Such protocols were used in the present study, in two separate geographic sites, resulting in two well-defined, APN-prone patient populations. We found an increased frequency of *CXCR1* sequence variants in both groups. The pediatric group had been followed from the first known febrile UTI episode by our clinical team and the adults were followed regularly from their first febrile UTI episode for a median of 30 years. The need for stringent clinical definitions is illustrated by a recent report, which failed to show a significant increase in variant 2 in patients with DMSA proven kidney infections [51]. In a second study, low CXCR1 expression was detected in 3/9 patients with childhood APN and two had SNPs in exon 2, but the numbers were small and no conclusions were drawn [52]. The stringent clinical follow up and large number of APN prone patients probably explains the high frequency of *CXCR1* variants and of reduced CXCR1 expression. On the other hand, the complicated clinical procedures illustrate the need for markers, which identify the risk patients already in connection with their first UTI episode. If this were possible, proper therapeutic interventions might be made and invasive diagnostic procedures restricted to patients at high risk, while those of lower risk might be spared. We are hopeful that the results of this study will be useful and that they might stimulate attempts to identify susceptible patients who might benefit from more intense diagnostic surveillance and therapeutic intervention.

## Supporting Information

Table S1 APN prone patients. Clinical data of the APN-prone pediatric patients included in the study.
(0.05 MB PDF)

Table S2 Amplification and sequencing primers used. Summary of the primers used for CXCR1 amplification and sequencing.
(0.03 MB PDF)

Table S3 Pyrosequencing CXCR1 genotyping primers and Vector construction. Summary of the primers used for genotyping by pyrosequencing and vector construction.
(0.04 MB PDF)
