# Supplementary material for: A Genetic Basis of Susceptibility to Acute Pyelonephritis
Source: PLoS One. 2007 Sep 5;2(9):e825. doi: 10.1371/journal.pone.0000825 (PMC1950574; doi:10.1371/journal.pone.0000825)
Supplement: Table S1 — APN prone patients. Clinical data of the APN-prone pediatric patients included in the study. (0.05 MB PDF) [file pone.0000825.s001.pdf]

# Table S1

***Table S1. APN prone patients***

| Patient no/gender | Age at first infection (years) | Diagnoses | | Reflux | | Comments | | DMSA scan |
|---|---|---|---|---|---|---|---|---|
| | | Index infection | Recurrence (no) | x / - ‡ | Grade | Sequence obtained | Variants† | Renal scar |
| 1/F | 2 | Py | - | + L | I | + | - | - |
| 2/F | 1 | Py | Cy (>5) | - | | + | - | - |
| 3/F* | 2 | Py | - | + L, R | III | + | - | - |
| 4/F* | 3 | Py | Cy (2) | - | | + | - | - |
| 5/F** | 1 | Py | Py (3) Cy (1) | - | | + | - | + |
| 6/F* | 5 | Py | - | + L, R | I, III | + | - | + |
| 7/F | <1 | Py | Py (>5) | + L | IV | + | - | + |
| 8/F | 6 | Py | - | - | | + | 1, 2 | - |
| 9/M | 5 | Py | Cy (1) | - | | + | 3 | - |
| 10/F | 5 | Py | ABU Cy (4) | - | | + | 4 | + |
| 11/F** | 5 | Py | Py (>5) | + R | Ureterocele | + | - | + |
| 12/M* | 9 | Py | Py (1-2) | + L, R | V, I | + | - | + |
| 13/F* | 1 | Py | ABU | + L, R | II | + | 1, 2 | - |
| 14/F | 1 | Py | - | - | | + | 1, 2 | - |
| 15/F | 2 | Py | Cy (1) | - | | + | - | + |
| 16/F | 3,5 | Py | Py (2) | + L | Op x3 | + | - | + |
| 17/F | <1 | Py | Py (>5) | + R, duplex | Duplex V, | + | - | + |
| 18/F* | 1 | Py | Cy (2) | - | | + | - | - |
| 19/F | <1 | Py | - | - | | + | 5 | - |
| 20/F | <1 | ABU | Py (1-2) Cy (1) | + R | V | + | 1, 2 | n.i. |
| 21/F | <1 | Py | - | - | | + | 1, 2 | n.i. |
| 22/M | <1 | Py | ABU | - | Hyndro-nephrosis | + | - | n.i. |
| 23/F | 5 | Py | Py, ABU | + R | III | + | - | n.i. |
| 24/F | <1 | Py | ABU | - | Hydro-nephrosis | + | 1, 2 | n.i. |

Py = pyelonephritis; Cy = cystitis; ABU = asymptomatic bacteriuria; L = Left, R = Right; ‡) The patients had uptake defects on DMSA scan; †) – = no detected genetic variants; n.i.= no information available. Patient was receiving *Trimetoprim or **Furadantin as prophylaxis.
