# Supplementary material for: A Genetic Basis of Susceptibility to Acute Pyelonephritis
Source: PLoS One. 2007 Sep 5;2(9):e825. doi: 10.1371/journal.pone.0000825 (PMC1950574; doi:10.1371/journal.pone.0000825)
Supplement: Table S2 — Amplification and sequencing primers used. Summary of the primers used for CXCR1 amplification and sequencing. (0.03 MB PDF) [file pone.0000825.s002.pdf]

Table S2

Table S2. Amplification and sequencing primers used.

Amplicon		Primers	
		PCR	Sequencing
A	Fwd	5’-GCTAACCAGCCAGACTCTGGGAGTG-3´	As PCR-primer
	Rev	5’-GGCGAAGGATTTGACTTACAGCAG-3´	As PCR-primer
B	Fwd	5’-GTAGAGATTGAGTGCCTGCTGTTC-3´	As PCR-primer
	Rev	5’-GTGGCTTCCTACCTGAAGCACCG-3´	As PCR-primer
1	Fwd	5’-AAAGAAGACCAGGTTGGAGGCAAAG-3’	5’-GGCAAAGGAGGAAGGGGAGC-3’
	Rev	5’-AGCTAGAGGGCACTTGATGAATAAACG-3’	5’-GGAAATAAGGAAACCACATCAGATGACAC-3’
2	Fwd	5’-TCATATGGACCCTGGCAGTCTCTAATC-3’	5’-TGTGATCCCTTGACTTAAACTGTTCTTCC-3’
	Rev	5’-AATCGAGGAAGACAGAATCATCCACC-3’	5’-TCCACCTCTCATTTGCTGCCAG-3’
3	Fwd	5’-AGTGAAAATGCAAATACTGGTAGGGAGG-3’	5’-CTGGTAGGGAGGGAACACACTAAAATG-3’
	Rev	5’-ACCCAGATGGCCCCTAAAGAGC-3’	5’-CCTCAAAGCTCCAGGACTCTCCAG-3’
4	Fwd	5’-CAAAGGCTGGGATTACACACGAAAC-3’	5’-CAGGATGGAATGAAGACTGGATGC-3’
	Rev	5’-CTGTGTCTGTGCATTTGGATGTGG-3’	5’-CATTTGGATGTGGGGACCAGG-3’
5	Fwd	5’-GGGAACAGGTTTGCCTTCTTGC-3’	5’-TTTGCTTAATGCTGGCCCTTCC-3’
	Rev	5’-GTCTCAGTTTCTAGCATACAGGGGCTG-3’	5’-GGGGCTGTAATCTTCATCTGCAGG-3’
6	Fwd	5’-CCTTCCAGTTAGATCAAACCATTGCTG-3’	5’-CAAACCATTGCTGAAACTGAAGAGGAC-3’
	Rev	5’-CCATCCGCCATTTTGCTGTG-3’	5’-CGCCATTTTGCTGTGTCATTTCC-3’
7	Fwd	5’-CCTTCTTCCTTTTCCGCCAGG-3’	5’-CCAGGCTTACCATCCAAACAATTCC-3’
	Rev	5’-AGAGCCAGATCACCTTCCACACAC-3’	5’-ACCTCAGGGTGTTGGTTATTCTTTCC-3’
8	Fwd	5’-CTCTTCCAACCTCTGAAAACCATCG-3’	5’-CTCTGAAAACCATCGATGAAGGAATATC-3’
	Rev	5’-TCTGGCTTCCAAACCCTCTTTCTC-3’	5’-CTCTTTCTCTTTGTTCATCCACCACC-3’
9	Fwd	5’-GTTTTCCCATCTCAGGTGTGTTGC-3’	5’-GGAGACATTGAGGCAGGCACTG-3’
	Rev	5’-GATCTTCCTTGGCCAGGGGTATG-3’	5’-GGGGTATGGGCATGAACAGGAG-3’
10	Fwd	5’-GCATTGTTGGCTGTTCCTG-3’	5’-AGTAGAATGGGGGCAGCAC-3’
	Rev	5’-TCCTCACTTGGGACATTTTTACAC-3’	5’-GGAGGCTGAAAGCGGAC-3’
