# Supplementary material for: A Genetic Basis of Susceptibility to Acute Pyelonephritis
Source: PLoS One. 2007 Sep 5;2(9):e825. doi: 10.1371/journal.pone.0000825 (PMC1950574; doi:10.1371/journal.pone.0000825)
Supplement: Table S3 — Pyrosequencing CXCR1 genotyping primers and Vector construction. Summary of the primers used for genotyping by pyrosequencing and vector construction. (0.04 MB PDF) [file pone.0000825.s003.pdf]

Table S3

Table S3. Pyrosequencing *CXCR1* genotyping primers and Vector construction

Amplicon		Primers: PCR	Primers: PCR-biotinylated
Var.1	Fwd	5’-TCATATGGACCCTGGCAGTCTCTAATC-3’	5’-CCGCTTCCTC GTGACC-Bio -3’
	Rev	5’-GCGAGGAAGACAGAATCATCCACC -3’	5’-GCTGTCCTCT TAACTC-3’
Var.2	Fwd	5’-CCTTCTTCCTTTTCCGCCAGG-3’	5’-CTGTCGTCCTCATCTTCCTG-Bio -3’
	Rev	5’-ACGCGCCAGATCACCTTCCACACAC -3’	5’-CGATGAAGGCGTAGATGATG -3’
Var.3/4	Fwd	5’-CTCTTCCAACCTCTGAAAACCATCG-3’	5’-CACACCAACCTTCTGAGGAG-Bio -3’
	Rev	5’-CGCTGGCTTCCAAACCCTCTTTCTC-3’	5’-CTCTCATCTAATGTCAGATTCG -3’
Var.5	Fwd	5’-GTTTTCCCATCTCAGGTGTGTTGC-3’	5’-GTGCAGCCACCAGTCCATTGGG-Bio -3’
	Rev	5’-CGATCTTCCTTGGCCAGGGGTATG -3’	5’-CGATCTTCCTTGGCCAGGGGTATG -3’

Sequencing

		Sequencing
Var.1	Rev	5’-GCTAACTCCATGTATGAGTG-3’
Var.2	Rev	5’-TTGCGGCGCTCAC -3’
Var.3/4	Rev	5’-AGTGGGTTAAAGATGTGA -3’
Var.5	Rev	5’-TCCACAGGGACAAGC-3’

Vector construction primers

CXCR1AML1wtFWD/CXCR1AML1wtREV	5´ **TCGAGCCTCTCTTGTGACCACCACTCATACATGGAG-3’**
	**5’-GATCCTCCATGTATGAGTGGTGGTCACAAGAGAGGC-3’**
CXCR1AML1SNP1FWD/CXCR1AML1SNP1REV	**5’- TCGAGCCTCTCTTGTGACCAGCACTCATACATGGAG-3’**
	**5’- GATCCTCCATGTATGAGTGCTGGTCACAAGAGAGGC-3’**
